# Effect of partial selfing and polygenic selection on establishment in a new habitat

**DOI:** 10.1111/evo.13812

**Published:** 2019-08-16

**Authors:** Himani Sachdeva

**Affiliations:** ^1^ Institute of Science and Technology Austria (IST Austria) Klosterneuburg 3400 Austria

**Keywords:** Adaptation, eco‐evolutionary models, inbreeding depression, partial selfing, polygenic architecture

## Abstract

This article analyzes how partial selfing in a large source population influences its ability to colonize a new habitat via the introduction of a few founder individuals. Founders experience inbreeding depression due to partially recessive deleterious alleles as well as maladaptation to the new environment due to selection on a large number of additive loci. I first introduce a simplified version of the inbreeding history model to characterize mutation‐selection balance in a large, partially selfing source population under selection involving multiple nonidentical loci. I then use individual‐based simulations to study the eco‐evolutionary dynamics of founders establishing in the new habitat under a model of hard selection. The study explores how selfing rate shapes establishment probabilities of founders via effects on both inbreeding depression and adaptability to the new environment, and also distinguishes the effects of selfing on the initial fitness of founders from its effects on the long‐term adaptive response of the populations they found. A high rate of (but not complete) selfing is found to aid establishment over a wide range of parameters, even in the absence of mate limitation. The sensitivity of the results to assumptions about the nature of polygenic selection is discussed.

Peripheral habitats such as islands and geographic range limits present demographic and adaptive challenges to the establishment of new populations (Kawecki 2008). Natural habitats often span environmental gradients, resulting in different selection pressures at the core and peripheries of the habitat. Peripheral habitats may also be subject to asymmetric gene flow, resulting in swamping, maladaptation, and the emergence of “demographic sinks” (Bridle and Vines 2006). Alternatively, habitats colonized by a single long‐distance dispersal event may be effectively isolated from the core, such that the establishing population is strongly influenced by founder effects and prone to stochastic extinction. Other challenges stem from low population density during the initial phases of establishment. This results in increased inbreeding and associated fitness costs, while also rendering the population vulnerable to mate limitation and demographic Allee effects (Courchamp et al. 1999).

Several empirical studies have suggested a causal link between the mating system of a population and its establishment success in a new habitat. In a highly influential paper, Baker (1955) hypothesized that self‐fertilizing species should be more adept at long‐distance colonization, and presented evidence for the overrepresentation of selfers on islands in comparison to the mainland. Subsequent work has revealed other examples of this general pattern (Barrett 1996; Grossenbacher et al. 2017), but also important exceptions, notably the abundance of dioecious plants on the Hawaiian archipelago (Carlquist 1966).

Arguments linking selfing to colonizing ability typically invoke reduced mate limitation in selfing populations (Baker 1955). Selfing, or more generally uniparental reproduction, provides *reproductive assurance*, allowing colonizers to survive the initial low‐density phase (Pannell et al. 2015). However, mating systems also affect other aspects of establishment—complete or partial selfing changes the average heterozygosity along the genome, the extent of linkage and identity disequilibrium (ID) between loci under selection, and the amount of genetic variation in the population. These characteristics of the source population influence its adaptive potential in a new habitat, as well as the extent of fitness loss (due to inbreeding) during the establishment bottleneck. Further, mating systems modulate outbreeding depression in the establishing population in the face of recurrent, maladaptive gene flow from the core habitat and thus, may themselves evolve under selection during establishment.

Given the many and possibly conflicting effects of mating system on establishment, theoretical models can play a crucial role in clarifying the range of environmental conditions and genetic parameters for which mating strategies such as increased selfing augment establishment success (Glémin and Ronfort 2013; Uecker 2017). An important challenge is to integrate polygenic architectures that often underlie adaptation into eco‐evolutionary models that consider how population size and genotypic frequencies coevolve.

Most theoretical work on polygenic adaptation during range expansions or the colonization of new habitats has focused on *randomly mating* populations (Kirkpatrick and Barton 1997; Polechova and Barton 2015; Tufto 2001; Barton and Etheridge 2018). These models give insight into whether and how interactions and associations between loci—generated either by selection or due to mixing of diverged populations—impact evolutionary dynamics during establishment.

However, selfing and other forms of nonrandom mating also generate strong multilocus associations, which have several effects on a population under selection. First, correlations between homozygosity at different loci cause most deleterious alleles to be masked from selection in outcrossing and weakly selfing populations, but efficiently purged at higher selfing rates. Thus, allele frequencies and inbreeding depression exhibit a nonlinear dependence on the selfing rate, especially when deleterious alleles are nearly recessive and the total mutation rate is high (Lande and Schemske 1985; Lande et al. 1994). Second, selfing reduces heterozygosity and the within‐family variance of quantitative traits, while increasing their between‐family variance (Wright 1951). Although the precise effect of selfing on quantitative trait variation depends on the nature of selection and the genetic architecture of the selected trait (Charlesworth and Charlesworth 1995; Kelly 1999; Lande and Porcher 2015), adaptive response from quantitative variation is expected to be generally different in selfed versus outcrossed populations.

In this article, I investigate how selfing within a large source population (e.g., on a mainland) influences its ability to colonize a new habitat (such as an island) in a scenario where the establishing population experiences both inbreeding depression and maladaptation to the new habitat due to selection on a large number of loci. For simplicity, environmental adaptation and inbreeding depression are assumed to be affected by two *different* sets of unlinked loci. Alleles at the first set of loci have partially recessive effects and are unconditionally deleterious on both the mainland and the island. Alleles at the second set of loci have co‐dominant effects and additively determine a trait, which is under environment‐dependent selection. The environmental trait is assumed to be under directional selection on both the mainland and island, but in opposite directions. The implications of these assumptions are explored in detail in the Discussion section.

The study has two parts: I first use a simplified version of the inbreeding history model (IHM) (Kelly 2007) to characterize mutation‐selection balance involving non‐identical, unlinked loci under multiplicative selection in a large, partially selfing source population. The focus is on elucidating the extent to which associations between loci are explained by differences in *recent* selfed versus outcrossed ancestry of individuals.

In the second part, I explore how the genetic composition of a large source population influences establishment probabilities on the island, following the introduction of a few founder individuals from the source. Successful establishment requires that the population both survive a transient increase in genetic load (due to higher inbreeding in small populations that generates homozygous combinations of recessive alleles) and adapt (via a response from existing genetic variation or new mutations). The goal is to understand how selfing within the source population affects both these aspects of establishment, and explain the resulting dependence of establishment probabilities on selfing rate. Another goal is to distinguish the effect of selfing on the *initial fitness* of founders from its effect on how variable and inbred their descendants are, which determines the long‐term *adaptive potential* of the population.

The interplay between partial selfing and polygenic selection in large populations has been analyzed via different theoretical approaches (Kondrashov 1985; Charlesworth et al. 1990, 1991; Lande et al. 1994; Kelly 1999, 2007; Roze 2015; Lande and Porcher 2015; Abu‐Awad and Roze 2018). A key challenge is to find tractable and accurate approximations for the multilocus associations that emerge due to partial selfing even in the absence of linkage. Roze (2015) and Abu‐Awad and Roze (2018) derive analytical expressions for allele frequencies (under various selection models) by assuming that these are only affected by *pairwise* associations between loci. This analysis is thus applicable when selective interference between loci (and resultant multi‐locus disequilibria) are not too strong, but becomes inaccurate for genome‐wide mutation rates *U* much higher than typical selective effect hs of deleterious alleles (see fig. 4 in Roze 2015).

An interesting approach by Kelly (1999, 2007) classifies individuals according to their *selfing age*, that is, the number of generations of continuous selfing in the lineage leading up to the individual. The partially selfing population can then be viewed as a structured population consisting of groups or cohorts of individuals of different selfing ages. Kelly (2007) used this approach to model identical loci subject to partially recessive, deleterious mutations. He derived recursions for the mean and variance of (and the correlation between) the number of loci that are homozygous and heterozygous for the deleterious allele within each selfing age cohort by assuming that associations, that is, linkage and identity disequilibria *within* cohorts are weak. The underlying assumption is that in the absence of linkage and epistasis, inbreeding coefficients vary between individuals of a population mostly due to differences in (recent) selfing history.

The present work employs a simpler approximation that neglects disequilibria within cohorts altogether, but accounts for population‐wide disequilibria that emerge due to differences in average allele frequencies or average homozygosity between cohorts. This approximation is thus slightly less accurate than that of Kelly (2007), but has the advantage of yielding simpler recursions that are easily generalized to describe *nonidentical* loci. As shown below, ignoring associations within cohorts yields reasonably accurate predictions for allele frequencies, pairwise associations between loci, and mean fitness and inbreeding depression in the population across a range of parameters. This also allows us to predict the genetic composition of source populations with different selfing fractions, without directly simulating large numbers of individuals with many selected loci.

Although the effects of inbreeding *during* establishment have been studied in recent theory (Barton and Etheridge 2018), the implications of having systematic deviations from panmixia in the source population itself remain largely unexplored. Dornier et al. (2008) consider how inbreeding depression and Allee effects shape the establishment potential of partially selfing populations by assuming a fixed level of inbreeding depression. However, as demonstrated below, establishment success depends on the interplay between inbreeding depression and the fitness of founders, which are correlated in a complex way when the total genomic mutation rate is high. Moreover, establishment often involves adaptation to a new environment via response from quantitative genetic variation. Modeling source populations with complex genetic architectures and nonrandom mating is thus an important step toward understanding more realistic population establishment or evolutionary rescue scenarios.

As the focus is on understanding how selfing affects establishment probability via the genetic composition of founding individuals subject to polygenic selection, we will only model a single bout of migration. Thus, we do not investigate how selfing affects outbreeding depression or heterosis, which may, however, play a role when the establishing population is subject to continuous gene flow from a divergent source population. Further, the analysis will focus on *initial* establishment: this distinction is important, since selfing may have different effects in small and growing versus large and equilibrated populations. Finally, selfing rates on the island are assumed to be the same as in the source population. Thus, the model does not allow for mating system evolution or plasticity in the new habitat, which could, however, be important during the establishment of natural populations (Peterson and Kay 2014).

## Model and Methods

### SOURCE POPULATION

Consider a large, partially selfing source population with *N* diploid, hermaphroditic individuals. Each individual genome has $L_{A}$ loci (referred to as additive loci henceforth), which undergo mutation between two alternative alleles with *co‐dominant* effects, and $L_{R}$ loci (referred to as recessive loci), which undergo mutation to deleterious alleles with *partially recessive* effects. The codominant alleles contribute additively to a trait *z* under directional selection. All loci are unlinked, and there is no epistasis between loci. Mutation between the two alternative allelic states occurs at rates $\mu_{A}$ and $\mu_{R}$ per locus per generation for the additive and partially recessive loci, respectively.

Individual fitness is given by $W=\exp[-\beta_{0}\left(z-z_{\text{min}}\right)-s\sum_{i=1}^{L_{R}}\left(X_{i}+hY_{i}\right)]$. Here, the summation is over $L_{R}$ recessive loci, each of which has selective disadvantage *s* (when homozygous for the recessive allele) and dominance coefficient *h*, with h<1/2. The variables $X_{i}$ and $Y_{i}$ are equal to 1, respectively, if the individual is homozygous or heterozygous for the recessive allele at locus *i*, and zero otherwise. For simplicity, effect sizes are also assumed to be the same at each additive locus contributing to the trait *z*: alternative alleles make contributions −α/2 or α/2, where α is arbitrarily set to $1/\sqrt{L_{A}}$ according to the usual quantitative genetics convention. The trait value *z* thus ranges from $z_{\text{min}}=-\alpha L_{A}$ to $z_{\text{max}}=\alpha L_{A}$, and the strength of selection per allele at each additive locus is ${\tilde{s}}_{0}=\beta_{0}\alpha$. It will sometimes be convenient to use the negative log fitness G=−ln(W), that is, the genetic load associated with an individual, which is the sum of two components: $\beta_{0}\left(z-z_{\text{min}}\right)$ and $s\sum_{i=1}^{L_{R}}\left(X_{i}+hY_{i}\right)$.

Generations are assumed to be nonoverlapping. The life cycle in each generation consists of mutation, followed by selection, and then mating via partial self‐fertilization (in which a fraction $r_{s}$ of individuals self). Because fitness is *multiplicative* across both types of loci, and loci are unlinked, there should be no multilocus associations in a sufficiently large population that is either purely outcrossing ($r_{s}=0$) or purely selfing ($r_{s}=1$). However, partial selfing ($0<r_{s}<1$) generates associations between allelic states (linkage disequilibrium [LD]) as well as between homozygosity (ID) at different loci even in the absence of epistasis, linkage, and drift (Weir and Cockerham 1973).

#### Identity and Linkage Equilibrium within Cohorts (ILEC) approximation

Associations arise due to differences in selfing histories and the resultant variation in homozygosity across individuals in a partially selfing population. Following Kelly (2007), we can define the *selfing age* of an individual as the number of generations back to its most recent outcrossing ancestor, or equivalently, the number of generations of continuous selfing in the lineage leading up to the individual. Thus, an individual produced by outcrossing in the present generation has selfing age 0, the selfed offspring of a parent produced via outcrossing in the previous generation has selfing age 1, and so on. Individuals with higher selfing ages have higher homozygosity on average.

Let the proportion of individuals with selfing age *i* be $f_{i}$, and the average frequency of homozygous loci (with two “1” alleles) among these individuals be $p_{11}^{\left(i\right)}$. By assuming that genotypes of different loci are uncorrelated *within* any cohort, we can express the ID between a pair of loci (of the same type) across the whole population as (Supporting Information):

(1) $${\left(ID\right)}_{\text{pair}}=\left[\sum_{i}\sum_{j<i}f_{i}f_{j}{\left[p_{11}^{\left(i\right)}-p_{11}^{\left(j\right)}\right]}^{2}\right]/p^{2}{\left(1-p\right)}^{2}$$

Here, the double summation is over all possible pairs of selfing ages. The normalization (denominator) term involves *p*, the population‐wide frequency of the “1” allele. Thus, population‐wide disequilibria arise due to the presence of cohorts with different average homozygosities and allele frequencies per locus, even when there are no associations within cohorts.

In general, each cohort is itself characterized by some population structure— for instance, the cohort with selfing age zero consists of outcrossed offspring of parents with diverse selfing histories (and hence slightly different allele frequencies), which generates LD within this cohort. However, the present approximation neglects all such multilocus associations (LD and ID) within a cohort. Then the state of the population is completely specified by the fraction $f_{i}$ of individuals belonging to cohort *i*, the frequencies of homozygous and heterozygous additive loci (denoted by $p_{11,A}^{\left(i\right)}$ and $p_{01,A}^{\left(i\right)}$, respectively) within the ith cohort, and the corresponding frequencies $p_{11,R}^{\left(i\right)}$ and $p_{01,R}^{\left(i\right)}$ for partially recessive loci. This will be referred to as ILEC approximation to distinguish it from the IHM introduced by Kelly (2007). Note that the latter also accounts for weak, pairwise disequilibria within cohorts.

Under the ILEC approximation, the evolution of the partially selfing population is described by specifying how the proportions $f_{i}$ and the frequencies $p_{11,A}^{\left(i\right)}$, $p_{01,A}^{\left(i\right)}$, $p_{11,R}^{\left(i\right)}$, and $p_{01,R}^{\left(i\right)}$ change due to mutation, selection, and partial selfing in each generation (Supporting Information Section S1 ). These *deterministic* equations ignore allele frequency changes due to drift, as well as stochastic fluctuations of the proportions $f_{i}$, and are thus only applicable for large source populations. These equations are iterated until equilibrium is attained. The equilibrium frequencies within each cohort and the corresponding fractions $f_{i}$ yield all population‐wide disequilibria (e.g., eq. 1, see also Supporting Information), as well as the full fitness distribution in the population, under the ILEC assumption.

#### Individual‐based simulations

The key assumption underlying the ILEC approximation is that a single round of outcrossing is sufficient to erase most associations between loci (within the outcrossed cohort) and that residual associations can be ignored for prediction of population attributes. This assumption is tested by simulating large populations for various parameter combinations.

Simulations are initialized by independently choosing the genotype at each locus for each of the *N* individuals. The population is evolved in discrete generations as follows—first, all individuals undergo mutation, where the allelic state of each locus is flipped (0↔1) with probability $\mu_{R}$ for a recessive locus and probability $\mu_{A}$ for an additive locus. *N* individuals are then chosen for mating by sampling from the population (with replacement) with weights proportional to individual fitness. Each individual is allowed to self with probability $r_{s}$ or outcross with probability $1-r_{s}$. For outcrossing individuals, the mating partner is chosen as before by fitness‐weighted sampling. All parental individuals produce gametes via free recombination of their diploid genomes. Selfed offspring are then created by pairing gametes from the same individual and outcrossed offspring by pairing gametes from the two (different) parental individuals.

**Figure 1:**
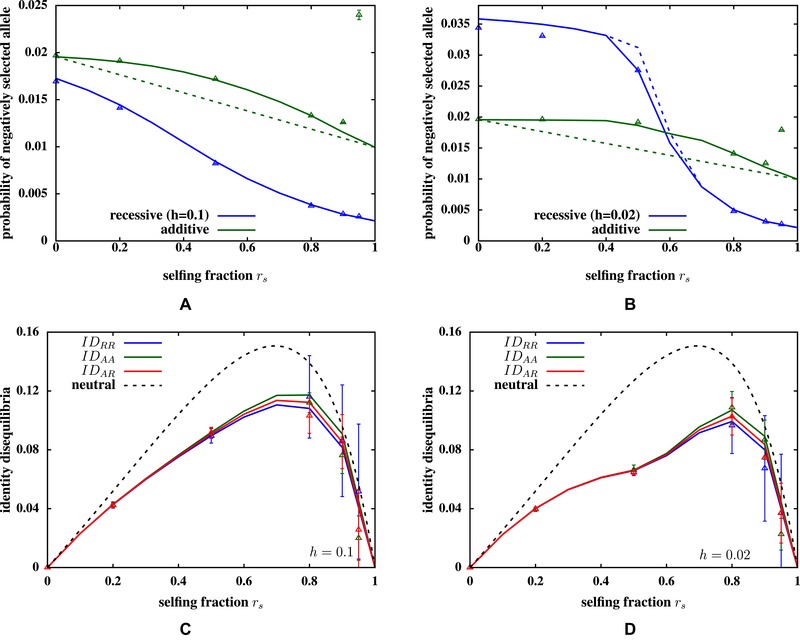
(A and B) Probability of finding a deleterious allele at a partially recessive (blue) or additive (green) locus versus selfing fraction $r_{s}$, when the genome has $L_{A}=1000$ additive and $L_{R}=5000$ partially recessive loci. The dominance coefficient of partially recessive alleles is h=0.1 in (A), and h=0.02 in (B). All loci are unlinked. Predictions of the ILEC approximation (solid lines) agree closely with results from simulations of N=10,000 individuals (triangles). Dashed lines are the corresponding allele frequencies (obtained from the ILEC approximation) when only one type of locus is present—thus, the green dashed line represents additive allele frequencies in a genome with 1000 additive loci (and no recessive loci). Presence of unlinked deleterious recessive mutations inflates the frequency of the unfavorable additive allele (dashed vs. solid green lines), especially for intermediate $r_{s}$. However, the frequency of recessive alleles is not strongly affected by unlinked additive alleles (dashed and solid blue lines are indistinguishable in (A)). Various pairwise identity disequilibria (ID) versus $r_{s}$, for h=0.1 (C) and h=0.02 (D). Green, blue, and red solid lines show the ILEC predictions for ID between two additive loci (${\text{ID}}_{\text{AA}}$), or two recessive loci (${\text{ID}}_{\text{RR}}$), or between an additive and a recessive locus (${\text{ID}}_{\text{AR}}$); triangles show the corresponding disequilibria, as obtained from individual‐based simulations of a population with *N* = 10,000. Dashed black line shows the neutral expectation for ID, as derived by Weir and Cockerham (1973). The mutation rate per locus is $\mu_{A}=\mu_{R}=10^{-4}$; selection against partially recessive deleterious alleles (in the homozygous state) is s=0.05 and against each additive allele is ${\tilde{s}}_{0}=\beta_{0}\alpha =0.005$.

**Figure 2:**
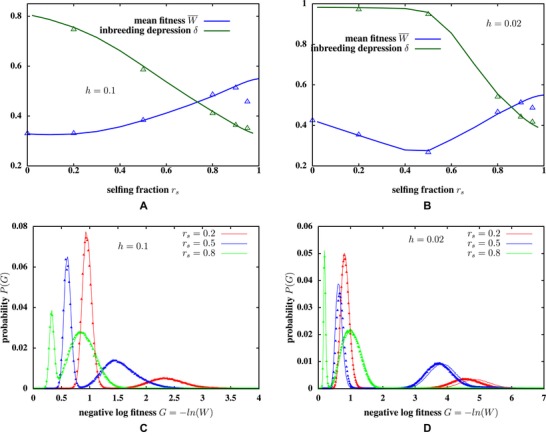
(A and B) Mean population fitness $\bar{W}$ and inbreeding depression δ versus selfing fraction $r_{s}$, when the genome has $L_{A}=1000$ additive and $L_{R}=5000$ partially recessive loci. The dominance coefficient of partially recessive alleles is h=0.1 in (A), and h=0.02 in (B). Solid lines represent predictions of the ILEC approximation, triangles depict results from simulations of N=10,000 individuals. (C and D) Comparison of simulation results (triangles) and ILEC predictions (lines) for the probability distributions of genetic load *G* (defined as negative log fitness G=−ln(W)) in the source population. The plots show load distributions for three different selfing fractions: $r_{s}=0.2,0.5,0.8$, and for two different dominance values of the recessive allele: h=0.1 (C) and h=0.02 (D). The distribution of *G* is bimodal due to significantly higher number of homozygous deleterious, recessive alleles in the genomes of selfed versus outcrossed offspring. Mutation rates per locus are $\mu_{A}=\mu_{R}=10^{-4}$, selection against partially recessive deleterious alleles (in the homozygous state) is s=0.05 and the strength of selection per additive allele is ${\tilde{s}}_{0}=\beta_{0}\alpha =0.005$.

The population is evolved for a few thousand generations until allele frequencies and disequilibria attain stationary values. For each set of parameters, reliable estimates of various quantities of interest are obtained by averaging over several replicates. All statistics are measured at the end of the generation. Comparisons with individual‐based simulations show that the ILEC approximation predicts detailed attributes of the source population such as pairwise disequilibria between loci, as well as the *distribution* of genetic load among individuals with reasonable accuracy, except when $r_{s}$ is close to 1 (Figs. 1 and 2).

### POPULATION ESTABLISHMENT IN THE NEW HABITAT

In the second part of the article, I investigate how founders from source populations with different selfing fractions colonize a new environment. As establishment typically involves a few individuals and proceeds via a small population phase, we cannot use the deterministic ILEC approximation and must simulate individuals to explicitly account for drift and demographic stochasticity.

However, founders from a large source population can still be drawn using the ILEC approximation: each founder is assigned a selfing age *i* with probability $f_{i}$, the proportion of individuals in cohort *i* in the source. The proportions $\left\{f_{j}\right\}$ depend on selection, dominance and mutation parameters, and the selfing rate in the source population, and are obtained from the ILEC approximation, as described above (see also Supporting Information). Then, each of the $L_{A}$ additive loci in the founder genome is independently assigned one of three possible genotypes: 00, 01/10, or 11 with probabilities $1-p_{01,A}^{\left(i\right)}-p_{11,A}^{\left(i\right)}$, $p_{01,A}^{\left(i\right)}$, or $p_{11,A}^{\left(i\right)}$ respectively. Here, $p_{01,A}^{\left(i\right)}$ and $p_{11,A}^{\left(i\right)}$ denote the frequency of additive loci that are heterozygous and homozygous for the “1” allele, within the ith cohort. Recessive loci are assigned genotypes similarly, that is, based on the equilibrium heterozygote and homozygote frequencies $p_{01,R}^{\left(i\right)}$ and $p_{11,R}^{\left(i\right)}$ in cohort *i*. Choosing genotypes independently at each locus reflects the assumption that there are no associations between loci within a cohort with a given selfing age.

Establishment is initiated by a single founder event in which *N*_0_ individuals from the source population are introduced all at once into the new habitat. There is no subsequent immigration. The direction of selection on the additive trait is reversed in the new habitat (with respect to the source population), such that individual fitness in the new habitat is: $\exp[-\beta_{1}\left(z_{\text{max}}-z\right)-s\sum_{i=1}^{L_{R}}\left(X_{i}+hY_{i}\right)]$, where β_1_ is positive, and is typically different from β_0_, the selection strength in the source. Contrast this with the fitness function in the source population: although partially recessive alleles are unconditionally deleterious in both habitats, different additive alleles are favored in the new habitat versus the source. Thus, additive alleles have *environment‐dependent* fitness effects. The establishing population is subject to *hard* selection in the new habitat, such that mean fitness influences population size.

Population establishment is studied via individual‐based simulations. These are initialized by randomly sampling *N*_0_ founder genomes from the source population, as described above. Mutation is implemented as before. Hard selection is enforced by assuming that the total number $N_{t+1}$ of offspring produced in generation t+1 is a Poisson‐distributed random variable with mean given by $\exp\left[r_{0}\left(1-N_{t}/K\right)\right]\bar{W}$. Here, *r*_0_ is the intrinsic rate of growth of the population, $N_{t}$ is the number of individuals prior to selection, *K* is the carrying capacity of the new habitat, and $\bar{W}$ is the mean population fitness, obtained by averaging over the fitness of all $N_{t}$ individuals.

Each of the $N_{t+1}$ offspring is assumed to be produced via selfing (with probability $r_{s}$) or outcrossing (probability $1-r_{s}$). One (or two) parent(s) of each selfed (or outcrossed) offspring is chosen from among the $N_{t}$ individuals by sampling with weights proportional to fitness. Note that if $N_{t}$ is small, then the same individual may be drawn both times while sampling the two parents of an outcrossed offspring. Thus, the realized selfing fraction may be much higher than $r_{s}$—being 1 if there is a single individual in the parental generation, and approaching $r_{s}$ as population size increases. Gametes are generated via free recombination, and paired to produce the next generation of individuals, as in the source population.

To assess the colonization potential of a source population, 10^3^ − 10^5^ independent colonization events are simulated. Each event involves *N*_0_ founders, independently sampled from the source. Probability of establishment in the new habitat is then computed as the fraction of “successful” establishment events among these. Establishment is considered successful if the population size is at least K/10 individuals, a certain number of (here 100) generations after the founder event.

##### Code Availability

All simulation codes associated with this manuscript can be found at: https://doi.org/10.5061/dryad.8tp0900.

## Results

### MUTATION‐SELECTION BALANCE IN THE SOURCE POPULATION: ILEC APPROXIMATION

We will first analyze attributes of a large source population (neglecting drift). Figure 1A and 1B shows the probability of finding a negatively selected allele at a locus (of each type), in an example where fitness is affected by both partially recessive and additive loci. Probabilities obtained from simulations of 10, 000 individuals are in close agreement with allele frequencies predicted by the ILEC approximation for both h=0.1 and h=0.02, across a range of selfing fractions.

The high rate of recessive mutations ($U_{R}=2\mu_{R}L_{R}=1$) relative to the (weak) selective effect per allele ($U_{R}/hs$ equal to 200 and 1000 in Fig. 1A and 1B, respectively) results in the segregation of a large number of recessive alleles. This generates substantial fitness differences between selfed and outcrossed individuals, especially in weakly selfing populations (see also Fig. 2D). Thus, selfers tend to be significantly underrepresented (relative to the selfing fraction $r_{s}$) among parents of the next generation of offspring. This implies that most deleterious alleles are masked from selection, because selection is less effective within the outcrossing cohort as compared to selfing cohorts, especially for low $r_{s}$ and when the average heterozygosity is high. As a consequence of this kind of *selective interference* between alleles, deleterious alleles are purged efficiently only at high selfing fractions, when selfed individuals make a nonnegligible genetic contribution to the next generation (Lande et al. 1994). The ILEC approximation captures the threshold selfing fraction, beyond which purging is effective, with reasonable accuracy (Fig. 1B), unlike calculations that only account for pairwise disequilibria (Roze 2015).

A related effect is observed at additive loci, where the frequency of unfavorable alleles is *inflated* by unlinked deleterious recessives segregating in the population (in Fig. 1A and 1B, compare solid lines, which represent allele frequencies in a genome having both additive and recessive loci, with dashed lines that represent allele frequencies in a genome with only one type of locus). This is again a consequence of high inbreeding depression due to recessive alleles, which strongly reduces the contribution of selfed individuals to the next generation. As a result, effective selection against unfavorable additive alleles is weaker than it would be in the absence of recessive alleles. This effect is typically quite modest, and is most significant at intermediate $r_{s}$, for which the mean and variance of the additive trait may increase by as much as 20−25% due to unlinked deleterious recessive alleles.

The ILEC approximation breaks down for finite populations with selfing fractions close to 1. In such populations, the effective population sizes $N_{e}$ is strongly reduced with respect to the census size, when the total genomic mutation rate is high relative to selection, that is, U/s≫1 (Charlesworth et al. 1993; Kamran‐Disfani and Agrawal 2014). This results in significant Hill‐Robertson interference between negatively selected alleles even in populations as large as 10, 000 and the buildup of negative LD between selected alleles (Kamran‐Disfani and Agrawal 2014). Thus, weakly deleterious alleles tend to drift close to fixation (due to the reduced efficacy of selection), which causes the average number of deleterious alleles per genome, as observed in simulations to be much higher than the corresponding ILEC prediction (Fig. 1A and 1B, see also Supporting Information Section S2). This reduction in $N_{e}$ is not captured by the deterministic ILEC approximation.

The ILEC approximation also predicts identity and linkage disequilibria between alleles at different loci. Figure 1C and 1D compares pairwise ID obtained from simulations with the corresponding ILEC prediction, and shows that the approximation is highly accurate across a range of selfing fractions. Note that ID between two loci is largely insensitive to the type of locus, being the same for two additive (blue triangles) or two recessive (green) or an additive and a recessive locus (red), for a given $r_{s}$. Moreover, ID is significantly lower than the neutral expectation (Weir and Cockerham 1973), shown via dashed lines in Fig. 1C and 1D, as genotypes that are homozygous for deleterious alleles at multiple loci are disfavored by selection. As expected, ID is strongest for intermediate selfing fractions, for which populations are maximally structured, that is, have a wide distribution of selfing ages and inbreeding coefficients. Pairwise LD is found to be negligible for these parameters (except for $r_{s}∼1$) both in simulations and according to the ILEC prediction.

Figure 2A and 2B shows the ILEC prediction for the average population fitness $\bar{W}$ and the inbreeding depression δ, defined as $1-{\bar{W}}_{\text{self}}/{\bar{W}}_{\text{oc}}$ (where ${\bar{W}}_{\text{self}}$ or ${\bar{W}}_{\text{oc}}$ is the average fitness of a randomly chosen selfed or outcrossed individual). As before, there is good agreement between simulations (triangles) and ILEC predictions (lines) for various dominance values and selfing fractions, except close to $r_{s}=1$. Note that $\bar{W}$ and δ depend on the full genotypic distribution, and are thus affected by all disequilibria (at least when selective interference between loci is strong).

For h=0.02, population fitness is minimum at intermediate selfing fractions (Fig. 2B). An increase in $r_{s}$ reduces the frequency of deleterious alleles (which tends to increase fitness), while increasing the average homozygosity (which tends to reduce fitness). Because highly recessive alleles are effectively masked from selection at low selfing fractions in the selective interference ($U_{R}/hs\gg 1$) regime, the reduction in deleterious allele frequency with $r_{s}$ is quite modest (Fig. 1B). Thus, increased selfing reduces fitness at low $r_{s}$ primarily by generating excess homozygosity. The ineffectiveness of selection at low $r_{s}$ is also reflected in the fact that inbreeding depression declines only beyond a threshold $r_{s}$ (Fig. 2B).

We can also generate the *distribution* of load in the population using the ILEC approximation (see Supporting Information Section S1) and compare this with equilibrium distributions from individual‐based simulations (Fig. 2C and 2D). Here, load is simply negative log fitness $G=-\ln\left(W\right)=\beta_{0}\left(z-z_{\text{min}}\right)+s\sum_{i=1}^{L_{R}}\left(X_{i}+hY_{i}\right)$ and is the sum of two components, the first due to additive alleles that influence environmental adaptation and the second due to unconditionally deleterious recessive mutations. The ILEC prediction is very accurate for higher dominance (h=0.1) but slightly less so when alleles are more recessive (h=0.02).

A key feature of the load distribution is that it is bimodal, with outcrossed individuals having significantly lower load due to recessive alleles than individuals with one or more generation(s) of continuous selfing in their lineage. This difference is especially marked when alleles are highly recessive and selfing fractions small or intermediate. Although average homozygosity is different between cohorts with different nonzero selfing ages, these differences are comparable to the variance of homozygosity within a cohort. Thus, the load distributions of cohorts with different nonzero selfing ages overlap significantly, resulting in a single broad peak at high load (Fig. 2C and 2D).

### POPULATION ESTABLISHMENT IN THE NEW HABITAT

To understand how selfing influences establishment in the new habitat, it is useful to first analyze scenarios where genetic load is either only due to unconditionally deleterious recessive alleles or only due to locally maladaptive additive alleles, and then consider selection on both. We will investigate establishment for a range of selfing fractions from $r_{s}=0$ to $r_{s}=0.9$. Completely selfing ($r_{s}∼1$) source populations (for which drift is important and the ILEC approximation inaccurate) are not investigated, but are briefly considered in the Discussion section.

#### Establishment with environment‐independent selection on recessive alleles

In this scenario, selection acts only on recessive alleles that have selective effect *s* (when homozygous) and dominance value *h* at each locus. As *s* and *h* do not vary across environments, establishment does not involve adaptation to a new environmental optimum, but only requires that the establishing population purge the excess genetic load that arises from increased inbreeding just after colonization.

Because the *N*_0_ founder genomes are generated using the deterministic ILEC approximation, there is no identity by descent due to drift at t=0. I further consider only those parameters $U_{R}$, *s*, and *h* for which a large source population would be viable under hard selection (for the same intrinsic growth rate *r*_0_). This is ensured by testing that a population with $N_{0}=100$ individuals doubles with probability greater than 0.95 within 100 generations, for very large *K*. In principle, drift, stochastic fluctuations, and inbreeding may be significant for $N_{0}=100$, which makes this a rather conservative test for the viability of *large* populations.

**Figure 3:**
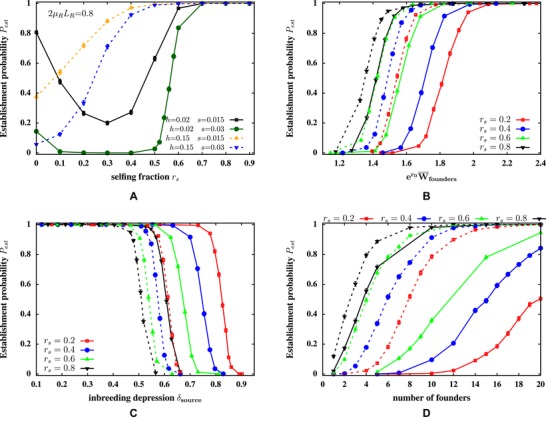
Establishment scenario where selection acts only on deleterious recessive alleles with environment‐independent selective effects. (A) Establishment probability $P_{\text{est}}$ versus selfing fraction $r_{s}$ for different selective effects *s* and dominance values *h* of deleterious mutations. $P_{\text{est}}$ is minimum at intermediate $r_{s}$ if genetic load is due to weakly selected, nearly recessive alleles, but increases monotonically with $r_{s}$ for larger *h*. Simulation parameters: $L_{R}=4000$, $\mu_{R}=10^{-4}$. (B and C) $P_{\text{est}}$ versus (B) initial average founder fitness $e^{r_{0}}{\bar{W}}_{\text{founders}}$ in the new habitat and (C) inbreeding depression $\delta_{\text{source}}$ in the source population, for different selfing fractions (represented by different colors) and different dominance coefficients (solid lines for h=0.02, dashed lines for h=0.1). The initial fitness of founders and inbreeding depression in the source are tuned by changing the total mutation rate $U_{R}$. For example, a population with $r_{s}=0.2$ and $U_{R}=0.00004$ has the same level of inbreeding depression ($\delta_{\text{source}}=0.5$) as a population with $r_{s}=0.8$ and $U_{R}=0.00011$ (with h=0.02). The number of founders is $N_{0}=10$. (D) $P_{\text{est}}$ versus the number of founders *N*_0_, for source populations with different $r_{s}$ and different dominance values (solid and dashed lines for h=0.02 and h=0.1, respectively). The mutation rate $U_{R}$ is chosen independently for each source population such that all populations have the same mean fitness $e^{r_{0}}{\bar{W}}_{\text{founders}}=1.6$. Simulation parameters in (B–D): $L_{R}=4000$, s=0.02. $P_{\text{est}}$ is the fraction of successful establishment events among 1000 independent simulation runs, each initialized by generating *N*_0_ founders from the source population using the ILEC approximation. Growth rate is $r_{0}=1.1$ and carrying capacity K=1000 in all figures.

Figure 3A shows how the establishment probability $P_{\text{est}}$ varies with the selfing fraction $r_{s}$ of the source population in an example with $N_{0}=10$ founders. Note how the dependence of $P_{\text{est}}$ on $r_{s}$ changes qualitatively with the recessivity and selective effect of alleles. $P_{\text{est}}$ is minimum for intermediate $r_{s}$ when genetic load is due to nearly recessive, weakly selected deleterious alleles. By contrast, when most segregating alleles are moderately recessive and deleterious, then $P_{\text{est}}$ increases monotonically with $r_{s}$ (ignoring $r_{s}∼1$ behavior).

As selection pressures on the mainland and island are identical, and we have only considered parameters for which a large source population would be viable under hard selection, failure to establish must arise solely from increased inbreeding in the new habitat, and cannot be due to low founder fitness. However, the extent to which inbreeding generates excess load and thus reduces establishment probability depends on both the initial fitness of founders and inbreeding depression in the source—even moderate inbreeding depression prevents establishment if the initial founder fitness is close to the threshold of viability ($e^{r_{0}}\bar{W}∼1$), while very fit founders ($e^{r_{0}}\bar{W}\gg 1$) would establish despite high inbreeding depression.

Thus, the complex dependence of $P_{\text{est}}$ on selfing fraction reflects the underlying dependence of both initial founder fitness and the magnitude of inbreeding depression on $r_{s}$. For highly recessive alleles and high genomic mutation rates $U_{R}$, fitness is minimum at intermediate selfing fractions in a large population (Fig. 2B). Moreover, inbreeding depression varies little with $r_{s}$ for weak selfing. Thus, founders with intermediate $r_{s}$ are least fit and experience similar levels of inbreeding depression as founders with $r_{s}=0$, which explains the minimum in $P_{\text{est}}$ at intermediate $r_{s}$ for h=0.02 (Fig. 3A). For less recessive alleles or lower mutation rates $U_{R}$, the average fitness of a large partially selfing population increases and the inbreeding depression decreases with $r_{s}$ (Fig. 2B). Thus, outcrossing populations are at maximum disadvantage, resulting in a monotonic increase in $P_{\text{est}}$ with $r_{s}$ for h=0.15 in Figure 3A.

To further disentangle the effects of founder fitness and inbreeding depression, we can plot $P_{\text{est}}$ as a function of $e^{r_{0}}{\bar{W}}_{\text{founders}}$ (Fig. 3B), where *r*_0_ is the intrinsic population growth rate, and ${\bar{W}}_{\text{founders}}$ is the average genetic fitness of founders in the new habitat, which, in this scenario, is just their mean fitness in the source. Figure 3B shows that $P_{\text{est}}$ becomes nonzero above a threshold founder fitness, which depends on both $r_{s}$ and the dominance coefficient *h*. For a given *h*, the threshold fitness required for establishment decreases with $r_{s}$. This simply reflects the fact that a strongly selfing population has lower heterozygosity, and hence, experiences less inbreeding load than a weakly selfing population with the same average fitness. Similarly, for a given selfing fraction, the threshold founder fitness is lower when mutations are less recessive (solid vs. dashed lines) due to the lower inbreeding depression and inbreeding load associated with higher values of *h*. The dependence on *h* is especially marked in weakly selfing populations.

It is also informative to plot $P_{\text{est}}$ versus inbreeding depression $\delta_{\text{source}}$ in the source population (Fig. 3C). As expected, establishment is successful only below a threshold value of $\delta_{\text{source}}$. Figure 3C further shows that this threshold for inbreeding depression is actually *lower* for populations with higher selfing fractions. This is due to the fact that a strongly selfing population must harbor more deleterious alleles on average and thus have lower fitness than a weakly selfing source population with the same level of inbreeding depression. Further, populations with the same selfing fraction $r_{s}$ and the same level of inbreeding depression have establishment probabilities, which depend on the recessivity of alleles contributing to inbreeding depression: $P_{est}$ is higher if alleles are *more* recessive (dashed vs. solid lines).

As the transient increase in inbreeding just after colonization depends crucially on the number *N*_0_ of founders, it is useful to consider how $P_{\text{est}}$ varies with *N*_0_ for founders drawn from source populations, which have the same mean fitness but different selfing fractions (Fig. 3D). Consistent with 3B, high rates of selfing allow for establishment with fewer founders, because of weaker inbreeding load during the establishment bottleneck. Further, $P_{\text{est}}$ increases more slowly with *N*_0_ for more recessive alleles, again because of higher inbreeding load associated with smaller values of *h*.

#### Establishment with environment‐dependent selection on additive alleles

**Figure 4:**
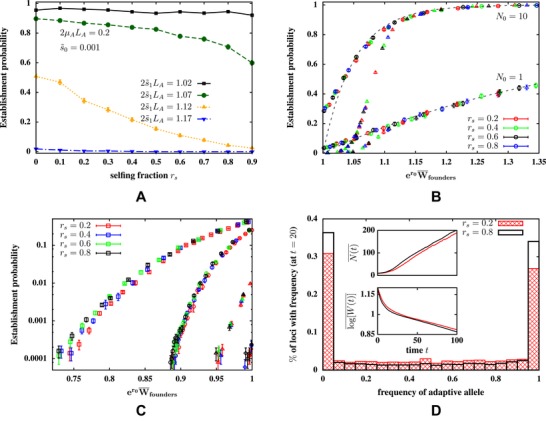
Establishment scenario where selection acts only on codominant alleles that additively determine a trait under environment‐dependent selection. (A) $P_{\text{est}}$ versus selfing fraction $r_{s}$ for different selection strengths (expressed as $2{\tilde{s}}_{1}L_{A}$) in the new habitat with $N_{0}=10$ founders. $P_{\text{est}}$ declines with increasing $r_{s}$ for intermediate $2{\tilde{s}}_{1}L_{A}$. Simulation parameters: $L_{A}=1000$, $\mu_{A}=10^{-4}$, ${\tilde{s}}_{0}=0.001$. (B and C) $P_{\text{est}}$ versus the initial founder fitness in the new habitat, for various $r_{s}$ (represented by different colors) and various selection parameters (represented by different symbols). Squares correspond to $L_{A}=100$, ${\tilde{s}}_{1}=10^{-2}$; circles to $L_{A}=100$, ${\tilde{s}}_{1}=6.25\times 10^{-3}$; triangles to $L_{A}=1000$, ${\tilde{s}}_{1}=10^{-3}$; diamonds to $L_{A}=1000$, ${\tilde{s}}_{1}=6.25\times 10^{-4}$; with $U_{A}=0.2$ in all four cases. Founder fitness is varied by changing the strength of selection in the source population, which changes the average frequency of alleles that are favored in the new habitat. Scenarios where the initial growth rate of founders in the new habitat is positive (B) or negative (C) are shown separately. (B) $P_{\text{est}}$ versus founder fitness for two selection parameters (represented by circles and triangles, see above) for two values of *N*_0_ is shown; the dashed lines represent predictions of a branching process approximation (see text). (C) $P_{\text{est}}$ versus founder fitness for four different selection parameters for $N_{0}=10$ is shown. It contrasts two different genetic architectures of the selected trait: 1000 loci that are weakly selected in the *new habitat* (triangles, diamonds) with 100 loci that are moderately selected (squares, circles). It also contrasts different levels of initial genetic variation: initially rare adaptive alleles (frequencies 0.02 − 0.12, circles and diamonds) versus adaptive alleles initially at intermediate frequency (0.28 − 0.45, squares and triangles). Initial genetic variation is changed by varying the strength of selection in the source population. (D) Main plot: Site frequency spectrum of adaptive alleles at additive loci, 20 generations after the founder event, for two groups of founders with the same mean fitness ($e^{r_{0}}{\bar{W}}_{\text{founders}}=0.896$), but low ($r_{s}=0.2$) or high ($r_{s}=0.8$) selfing fractions. Insets show average size $\bar{N(t)}$ (upper inset) and average load $\bar{ln[W(t)]}$ (lower inset) of the establishing population versus time *t* for the two selfing fractions. All quantities are calculated by averaging over those replicates in which the population establishes within 100 generations. Simulation parameters for (D): $L_{A}=100$, $U_{A}=0.2$, ${\tilde{s}}_{1}=0.01$. Intrinsic growth rate is $r_{0}=1.1$ and carrying capacity K=1000 in all figures.

Consider now a scenario where individuals only carry loci that mutate between alternative co‐dominant alleles, which additively determine a trait *z*. An individual with trait value *z* has fitness proportional to $\exp[-\beta_{0}\left(z-z_{\text{min}}\right)]$ in the source population, and $\exp[-\beta_{1}\left(z_{\text{max}}-z\right)]$ in the new habitat, where β_0_ and β_1_ are both positive. Thus, establishment is primarily constrained by maladaptation of the founders to the new environment, and is aided by the ability of the population to adapt from standing variation. Figure 4A shows how $P_{\text{est}}$ varies with the selfing fraction $r_{s}$, following a single colonization event with $N_{0}=10$ founders.

In the absence of deleterious recessive mutations, the frequency of the locally unfavorable additive allele is approximately $∼\left(\mu_{A}/2\tilde{s_{0}}\right)\left(2-r_{s}\right)$ in the source population (Ohta and Cockerham 1974; see also dashed lines in Fig. 1A and 1B). Thus, stronger selfing reduces the frequency of alleles that are selected against in the source and conversely, favored in the new habitat (where the direction of selection on the additive trait is reversed). Consequently, other parameters being equal, founder fitness in the new environment falls with $r_{s}$, which causes establishment probabilities to also decline with $r_{s}$ (Fig. 4A). This dependence on $r_{s}$ only arises close to a threshold selection strength (per allele) $\tilde{s_{1}}=\beta_{1}\alpha$, for which the genetic load of founders in the new habitat, given by $2\tilde{s_{1}}L_{A}\left[1-\left(\mu_{A}/2\tilde{s_{0}}\right)\left(2-r_{s}\right)\right]$, is comparable to the growth rate *r*_0_. Populations fail to establish, irrespective of selfing fraction, when selection in the new habitat is very strong ($2\tilde{s_{1}}L_{A}\gg r_{0}$). Conversely, for $2\tilde{s_{1}}L_{A}\ll r_{0}$, founders have high establishment success, irrespective of $r_{s}$.

As before, we can ask: does selfing influence establishment probabilities predominantly via its effects on founder fitness or on the rate of adaptation of the establishing population? Figure 4B and 4C show $P_{\text{est}}$ as a function of initial founder fitness, which is varied by changing the strength of selection in the source population, which changes the frequency of adaptive alleles among founders. Figure 4B and 4C represent, respectively, parameter combinations for which the initial growth rate of founders in the new habitat is positive ($e^{r_{0}}{\bar{W}}_{\text{founders}}>1$) or negative ($e^{r_{0}}{\bar{W}}_{\text{founders}}<1$). Different colors represent various selfing fractions; the different symbols correspond to different values of the selection coefficient ${\tilde{s}}_{1}$ (in the new habitat) and number of loci $L_{A}$ (see caption).

Note that for given $L_{A}$ and ${\tilde{s}}_{1}$, curves of various colors (corresponding to different $r_{s}$) more or less collapse onto each other (at least while $P_{\text{est}}$ is not too small). This suggests that selfing affects $P_{\text{est}}$ primarily via founder fitness. $P_{\text{est}}$ does, however, increase modestly with $r_{s}$ (for a given value of founder fitness) when $e^{r_{0}}{\bar{W}}_{\text{founders}}$ is significantly less than 1 and establishment correspondingly rare. This is most pronounced for moderately strong selection per locus and high initial standing variation (squares in Fig. 4C).

We can further investigate this weak dependence on $r_{s}$ by monitoring the site frequency spectrum (SFS) of adaptive alleles during establishment. Figure 4D compares the SFS within establishing populations initiated by two equally fit groups of founders with different selfing fractions ($r_{s}=0.2$ and 0.8). Higher selfing drives more adaptive alleles close to fixation, but also results in increased fixation of *maladaptive* alleles and depletion of intermediate‐frequency polymorphisms that contribute to ongoing adaptive response. These two countervailing effects thus cause the rate of adaptation and population growth to increase only weakly with the selfing fraction (inset, Fig. 4D).

Interestingly, Figure 4B and 4C reveal a marked dependence of establishment probabilities on the genetic architecture of the selected trait, when the initial growth rate of the population is negative or weakly positive. In particular, founders carrying adaptive alleles at a large number of *weakly* selected loci are less likely to establish than equally fit founders carrying adaptive alleles at a smaller number of *moderately* selected loci (circles vs. triangles or squares vs. diamonds in Fig. 4B and 4C). This is due to the fact that weakly selected alleles are rapidly eliminated by drift in the first few generations after the founding event, whereas moderately selected alleles tend to persist long enough for N(t) to become larger than 1/s, whence their frequencies increase in a sustained manner in response to selection.

Genetic architecture has very little effect on $P_{\text{est}}$ if the initial growth rate of founders is significantly positive. In this case, modeling initial establishment as a simple branching process with mean growth rate $e^{r_{0}}{\bar{W}}_{\text{founders}}-1$ yields an approximate expression for $P_{\text{est}}$, which closely matches simulations (dashed lines vs. points in Fig. 4B, see also Supporting Information Section S3). Thus, as expected, adaptation plays little role during initial establishment with highly fit founders.

Figure 4C also compares founders with low versus intermediate frequency adaptive alleles, which corresponds to low versus high additive variation (circles and triangles versus squares and diamonds). For given founder fitness and selfing fraction, $P_{\text{est}}$ is highest for founding cohorts in which additive variation is highest *and* selection per allele strongest (squares in Fig. 4C): then, establishment is moderately likely even for $e^{r_{0}}{\bar{W}}_{\text{founders}}\ll 1$. However, in general, additive genetic variation by itself does not predict establishment success: for instance, founders with high genetic variation arising from intermediate‐frequency polymorphisms at many weakly selected loci (triangles) are less likely to establish than equally fit founders with *lower* genetic variation due to low‐frequency alleles at fewer, moderately selected loci (diamonds). As before, this is explained by the rapid erosion of variation at weakly selected alleles in a small establishing population.

#### Establishment with selection on both types of alleles

**Figure 5:**
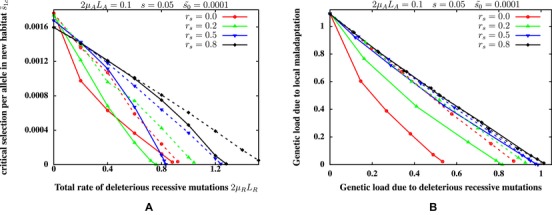
Phase diagrams showing parameter combinations for which the establishment probability $P_{\text{est}}$ is greater than 0.05, when individual genomes contain both partially recessive alleles with environment‐independent deleterious effects, and additive alleles with environment‐dependent effects. (A) Critical selection strength ${\tilde{s}}_{1,c}$ per additive allele in the new habitat versus the total mutation rate $U_{R}$ for partially recessive alleles, for different values of $r_{s}$ and *h*. For a given $U_{R}$, the establishment probability $P_{\text{est}}$ is greater than 0.05 only if ${\tilde{s}}_{1}<{\tilde{s}}_{1,c}$, that is, in the parameter regions below the corresponding line. (B) The maximum (average) genetic load due to environmental maladaptation that still allows for establishment (with $P_{est}>0.05$), as a function of the genetic load due to deleterious recessive alleles. Solid and dashed lines depict phase boundaries when deleterious alleles have dominance values h=0.02 and h=0.2, respectively; different colors show phase boundaries for different values of $r_{s}$. Establishment probabilities are obtained as the fraction of successful establishment events out of 1000 independent simulation runs, each initialized by sampling $N_{0}=10$ founders from the source population. Growth rate is $r_{0}=1.1$ and carrying capacity is K=1000. Other parameters are as follows: $L_{A}=500$, $\mu_{A}=10^{-4}$, $L_{R}=2000$, s=0.05, ${\tilde{s}}_{0}=0.0001$.

Finally, consider establishment scenarios with selection on both unconditionally deleterious, partially recessive alleles, and additive alleles with environment‐dependent effects. Figure 5 compares source populations with different total mutation rates $U_{R}$, and hence different magnitudes of genetic load due to partially recessive variants. For each $U_{R}$, we can find the critical environmental selection strength ${\tilde{s}}_{1,c}$ (per allele), such that $P_{\text{est}}$ is significant (greater than 0.05), as long as selection per allele in the new habitat is weaker than ${\tilde{s}}_{1,c}$ (Fig. 5A). A high value of ${\tilde{s}}_{1,c}$ signifies that founders can establish despite a large reversal of environmental selection in the new habitat.

As expected, for any $r_{s}$, the range of environmental selection strengths, to which a population can adapt, shrinks as $U_{R}$ increases. When $U_{R}$ is close to zero, outcrossing populations ($r_{s}∼0$) can adapt to slightly larger shifts than highly selfing populations ($r_{s}=0.8$). However, for even modest $U_{R}$, founders from the $r_{s}=0.8$ population establish over a larger range of ${\tilde{s}}_{1}$, due to the significantly lower deleterious recessive load and inbreeding depression in large, highly selfing populations. Note that this is true for both completely recessive (h=0.02, solid lines in Fig. 5A) and moderately recessive alleles (h=0.2, dotted lines).

We can also measure different components of the genetic load (negative log fitness −ln(W)) associated with founders for parameter combinations with $P_{\text{est}}>0.05$. The genetic load is the sum of two components—one arising from deleterious recessive mutations and the other from local maladaptation of the additive trait. The first component has average value $Ls[p_{11}^{R}+hp_{01}^{R}]$ (where $p_{11}^{R}$ and $p_{01}^{R}$ are the homozygote and heterozygote frequencies of the recessive allele in the source), and the second component has average value $\beta_{1}\left(z_{\text{max}}-\bar{z}\right)$ (where $\bar{z}$ is the population average of the additive trait in the source). Figure 5B depicts the values of these two components on a two dimensional phase plot: for instance, 10 founders drawn from a source population with $r_{s}=0$ and dominance h=0.02 of the recessive alleles, establish a viable colony with probability greater than 0.05, only for points (representing the two load components) lying below the solid red line.

Figure 5B shows that the total load is a good predictor of establishment success when deleterious mutations are less recessive and source populations strongly selfing. However, for weakly selfing populations, which suffer significant inbreeding depression due to highly recessive alleles, establishment success cannot be predicted on the basis of the total genetic load of founders, but requires a consideration of individual components of load. Thus, for low $r_{s}$ and *h*, the threshold total fitness required for establishment is significantly higher (i.e., maximum possible load significantly lower) for higher rates of deleterious recessive mutations.

## Discussion

Partial selfing is common in various hermaphroditic plant and animal taxa (Goodwillie et al. 2005; Jarne and Auld 2006), although estimating the exact fraction of species with intermediate selfing rates can be challenging (Igic and Kohn 2006). The extent of inbreeding depression in partial selfers may be similar to that in outcrossers, especially among long‐lived taxa such as gymnosperms that have high per‐generation mutation rates (Winn et al. 2011). This suggests a highly polygenic architecture of inbreeding depression, characterized by selective interference between recessive alleles (Lande et al. 1994), in many natural populations. Extensive work on the genetics of inbreeding depression points toward an important role of both highly recessive lethals and moderately recessive, weakly deleterious alleles (Charlesworth and Willis 2009). More generally, estimates in *Drosophila melanogaster* and *Saccharomyces cerevisiae* reveal a wide distribution of dominance values of deleterious alleles, with a mean of 0.1–0.2 (Agrawal and Whitlock 2011; Peters et al. 2003), and also suggest a negative correlation between the dominance values and selective effects of deleterious alleles (Charlesworth 1979).

The present study represents a preliminary attempt to understand how selfing and polygenic selective architectures together shape eco‐evolutionary dynamics during establishment in a new habitat. Our model makes several assumptions about the genetic architecture of the establishing population, whose implications are examined below. First, the model assumes that inbreeding depression and response to environmental selection are due to distinct, nonoverlapping sets of unlinked loci. Second, source populations are assumed to be in *deterministic* mutation‐selection balance, with a negligible role for drift. Further, the model assumes directional selection on the environment‐dependent trait. Under these assumptions, a high rate of selfing is found to facilitate establishment in several distinct ways.

### ESTABLISHMENT WITH UNCONDITIONALLY DELETERIOUS RECESSIVE ALLELES

Large populations harbor a substantial number of deleterious recessive alleles when the genomic rate of deleterious mutations $U_{R}$ is high relative to the typical selective effect hs. In this scenario, colonies founded by a few individuals may fail due to a kind of genetic Allee effect, wherein an increase in the fraction of selfed individuals (over and above $r_{s}$) or mating between related individuals depresses fitness, which reduces population size and further increases inbreeding, ultimately resulting in extinction. This effect is ameliorated if the source population is itself highly selfing and has low inbreeding depression, which gives highly selfing founders an advantage over equally fit, weakly selfing founders (Fig. 3B).

Our analysis points toward the difficulty of predicting establishment success solely on the basis of average founder fitness or based on inbreeding depression alone. In a partially selfing population with significant selective interference between loci, these two quantities bear no simple relationship to each other, unlike for $U_{R}/hs∼1$ (Bataillon and Kirkpatrick 2000). Founders with the same average fitness show different degrees of inbreeding depression δ that depend on *s*, *h*, $r_{s}$, and $U_{R}$. Conversely, founders drawn from populations with the same δ have different mean fitness and are thus affected by inbreeding load to different extents. In particular, the threshold fitness required for establishment is higher in selfing versus outcrossing founders with similar levels of inbreeding depression (Fig. 3D). This also suggests that including fitness and inbreeding depression estimates of continental progenitor species in meta‐analyses of island populations (Grossenbacher et al. 2017), wherever possible, may help to clarify the mixed evidence for Baker's law (1955).

The success of highly selfing founders in establishing despite the initial bottleneck hinges on the purging of deleterious variants in large source populations at high $r_{s}$. Purging is less effective, however, when the source population is itself small (Glémin 2003), as is often the case in human‐assisted re‐introduction of endangered species into new habitats. Understanding how the genetic composition of *small* source populations influences their establishment potential remains an important challenge in conservation biology.

The realized rate of selfing or biparental inbreeding during initial establishment depends crucially on the effective number of founders. In the present model, this is equal to or less than 2*N*_0_, for example, when a population is founded by *N*_0_ seeds of a diploid plant. Alternatively, in populations founded by *N*_0_ fertilized adults (carrying sperm from one or many fathers), the effective number of founders could be larger than 2*N*_0_. Importantly, our model does not consider true self‐incompatibility; thus, founders with $r_{s}=0$ can, nevertheless, self under mate limitation, resulting in a severe *genetic* Allee effect. If founders are obligate outcrossers, then the realized rate of selfing in the new habitat is zero, irrespective of *N*_0_. In this case, outcrossers suffer less from inbreeding depression during establishment, but are subject to a *demographic* Allee effect, wherein population growth rate is strictly zero for $N_{0}=1$. This leads to an even stronger advantage for self‐compatible founders for low *N*_0_.

### ESTABLISHMENT INVOLVING ENVIRONMENTAL ADAPTATION FROM ADDITIVE VARIATION

Selfing has a qualitatively different effect when establishment involves environmental adaptation from segregating variants or de novo mutation at additive loci. We have analyzed a specific scenario in which the direction of selection on the environmental trait is reversed in the new habitat, so that the response to selection involves rare or intermediate‐frequency variants. A key finding is that for highly polygenic architectures, with weak selection per locus, adaptive response is largely ineffective, so that initial establishment is essentially determined by the initial fitness advantage of founders. Thus, in this regime, selfing fraction influences $P_{\text{est}}$ only via founder fitness (Fig. 4B and 4C).

For moderate selection per locus (e.g., with less polygenic traits), adaptation plays a more significant role, allowing for establishment despite low founder fitness. In this regime, higher rates of selfing marginally increase establishment probabilities (for a given founder fitness). As a point of reference, note that the fixation probability of co‐dominant (h=1/2) alleles in a large population of constant size is predicted to be *independent* of selfing fraction (Caballero and Hill 1992; Glémin and Ronfort 2013). However, as evident from Figure 4D, selfing has a modest effect on selected variants in small, growing populations.

In an alternative scenario, where the environmental trait is under stabilizing selection toward different optima in the two habitats, selection on the establishing population would again (initially) be directional, as in the present model. However, adaptive response would now involve both high‐ and low‐frequency variants, and would be constrained to some extent by negative LD between adaptive variants. An additional complication is that, with strong stabilizing selection, co‐dominant alleles may also contribute to inbreeding depression: selfed cohorts have higher trait variance and lower fitness than outcrossed cohorts, and thus make a lower genetic contribution to the next generation. As a result, trait variance is predicted to be insensitive to selfing at low selfing rates but purged at higher selfing rates (Lande and Porcher 2015). Understanding how selfing affects establishment success in this scenario via effects on the genetic variation of the founding cohort, as well as on the rate of adaptation during the small population phase, is an interesting avenue for future work.

In general, for traits determined by many small‐effect loci, the genotypic values of the offspring of any two individuals are approximately normally distributed (Barton et al. 2017). In the absence of selfing, this allows for an economical description of the eco‐evolutionary dynamics of an establishing population within an infinitesimal framework (Barton and Etheridge 2018) using relatively few parameters such as the population size, the mean and variance of the trait under selection, and the distribution of inbreeding coefficients in the population. In principle, this framework can be extended to include selfing (although incorporating dominance within the infinitesimal model is more challenging). A key feature of selfing is that it *redistributes* variance (from within families to between families), unlike inbreeding due to small population sizes (considered by Barton and Etheridge 2018), which reduces both within and between family variance. Thus, the two forms of inbreeding should have qualitatively different effects on establishment.

### ESTABLISHMENT INVOLVING BOTH INBREEDING DEPRESSION AND ADAPTATION TO A NEW ENVIRONMENT

Under the assumptions of this model, highly selfing populations establish over a wider parameter region, especially when the total rate $U_{R}$ of deleterious mutations is large, and mutations highly recessive. Importantly, with low or intermediate $r_{s}$, establishment success depends not only on founder fitness, but also on what proportion of fitness loss is due to recessive alleles (Fig. 5B).

The present model makes the convenient assumption that the alleles that contribute to local adaptation and the alleles that are unconditionally deleterious are distinct and unlinked. In reality, variants that affect fitness are often highly pleiotropic. Thus, an alternative model would be one where each of many loci affect multiple traits under stabilizing selection. In this model, most alleles would be deleterious and have recessive effects on fitness *on average*, though with a wide variance of dominance coefficients (Manna et al. 2011). A shift in the selection optima for one or more traits in the new habitat would then necessitate a response from variants with a range of adaptive effects and contributions to inbreeding depression. This model could thus provide an alternative framework for investigating the effects of inbreeding depression and polygenic adaptive response on establishment.

The present model ignores linkage between selected alleles, which could, however, qualitatively change several of our conclusions. When loci are tightly linked, the effective rate of recombination and effective population size are significantly reduced, even in populations with $r_{s}$ not very close to 1, rendering selection against deleterious alleles ineffective (Kamran‐Disfani and Agrawal 2014). By the same token, linkage generates Hill–Robertson interference between *adaptive* alleles, which reduces the advantage selfers experience during adaptation from co‐dominant or even mildly recessive alleles (Hartfield and Glémin 2016). Finally, linkage increases hitchhiking of deleterious variants with adaptive alleles, especially in highly selfing populations, which reduces the fixation probability of even slightly recessive adaptive alleles (Hartfield and Glémin 2014; Kamran‐Disfani and Agrawal 2014). An interesting question is whether linkage could thus reduce the selfing fraction that is “optimal” for establishment in new habitats.

Although this study does not investigate establishment for $0.95<r_{s}<1$ (because of the difficulty of efficiently generating equilibrated source populations with selfing fractions in this range), we can venture some hypotheses on the establishment potential of completely selfing founders based on our analysis. For low values of U/s, when selective interference between loci is unimportant and population attributes largely predicted by the ILEC approximation across all selfing rates (see Supporting Information Section S2), $r_{s}∼1$ founders should establish with higher probability than equally fit founders with high (but not complete) selfing. This is because the latter suffer from higher inbreeding load during establishment due to segregation of recessive alleles, but exhibit similar initial adaptive response (which depends very weakly on $r_{s}$ for co‐dominant alleles). This is also consistent with observed behavior in Figure 5.

However, in the selective interference regime, completely selfing populations are much less fit than high $r_{s}$ populations with similar total mutation rates (Fig. 2C and 2D). Even when comparing founding cohorts with equal fitness, the significant reduction in effective population size close to $r_{s}∼1$ should severely constrain adaptive response in the establishing population, while also causing more deleterious alleles to fix. This suggests that $r_{s}∼1$ populations should have lower establishment success than highly selfing populations (with $r_{s}<1$) for U/s≫1. However, a more comprehensive analysis is required to confirm these hypotheses and understand eco‐evolutionary outcomes for $r_{s}∼1$ populations across different parameter regimes.

Our analysis focused on establishment after a single founder event. A natural extension is to study establishment with steady migration from the source (Barton and Etheridge 2018). Continual gene flow is expected to alleviate the high inbreeding load experienced by predominantly outcrossing populations via heterosis, and thus reduce the advantage of highly selfing founders during initial establishment. On the other hand, a highly selfing population, once established, might better withstand maladaptive gene flow from the mainland and experience less outbreeding depression. An interesting question is whether different mating strategies might be favored by natural selection during different phases of establishment.

For simplicity, the analysis included only two kinds of loci. However, the ILEC approximation provides a computationally frugal way of studying multiple loci with a distribution of selective and dominance effects. Understanding multilocus associations in terms of population structure arising from recent selfing history (Kelly 1999, 2007) is a powerful but relatively underutilized approach for studying partially selfing populations (although see Lande and Porcher 2015). Extending the ILEC (or similar) approximations to predict multilocus associations under more complex forms of selection can provide general insight into how the interaction between population structure and polygenic selection shapes the eco‐evolutionary dynamics of partially selfing populations.

Associate Editor: S. Glemin

Handling Editor: D. W. Hall

## Supporting information


**Figure 1**: Comparison of individual‐based simulations with ILEC predictions for high selfing fractions ($r_{s}$ = 0:95, 0:99 and 1), for genome‐wide mutation rate (A) *U* = 0:2 and (B) *U* = 1.
