# Supplementary material for: Effect of partial selfing and polygenic selection on establishment in a new habitat
Source: Evolution. 2019 Aug 16;73(9):1729–45. doi: 10.1111/evo.13812 (PMC6771878; doi:10.1111/evo.13812)
Supplement: Supplementary file 1 — Figure 1: Comparison of individual‐based simulations with ILEC predictions for high selfing fractions (rs = 0:95, 0:99 and 1), for genome‐wide mutation rate (A) U = 0:2 and (B) U = 1. [file EVO-73-1729-s001.pdf]

SUPPORTING INFORMATION FOR

# Effect of partial selfing and polygenic selection on establishment in a new habitat

## S1. Identity and Linkage Equilibrium within Cohorts (ILEC) approximation

This paper introduces the Identity and Linkage Equilibrium within Cohorts (ILEC) approximation, which is a simplified version of the Inbreeding History Model (Kelly , 2007). The basic idea is to approximate the state of a large, partially selfing population by neglecting all correlations between the allelic states and homozygosity of different loci, among individuals who have the same *selfing age*. The selfing age of an individual is defined as the number of generations back in time to its most recent outcrossing ancestor. Thus, an individual produced by an outcrossing event in the present generation has selfing age 0, an individual produced via selfing from a parent who was itself produced by an outcrossing event in the previous generation has selfing age 1, and so on.

Cohorts with higher selfing ages have higher average homozygosity; this results in more efficient selection against deleterious alleles in such cohorts, and consequently small allele

frequency differences between cohorts. However, the average fitness of a cohort tends to decrease with its selfing age (due to an increase in homozygosity), if most segregating alleles are recessive. Thus a partially selfing population can be viewed as a *structured* population consisting of cohorts with different selfing ages (or more generally different selfing histories) which contribute to the next generation in proportion to their fitness. A structured population has non-zero linkage and identity disequilibria, even when there are no associations between loci *within* sub-groups of the population, simply due to allele frequency or homozygosity differences between sub-groups. This is demonstrated below for the identity disequilibrium, i.e., the correlation in identity by descent, between a pair of identical loci.

For simplicity, consider the case where individuals have only one type of locus. Let us use $f_i$ to denote the fraction of individuals in the population with selfing age $i$, and $p_{11}^{(i)}$ to denote the average frequency of homozygous loci within the cohort with selfing age $i$. Assuming that the genotypes of different loci are uncorrelated within any cohort, the fraction of individuals in cohort $i$ who are homozygous for the 1 allele at two loci is $[p_{11}^{(i)}]^2$. Thus, the frequency of single-locus homozygotes across the whole population is $\sum_i f_i p_{11}^{(i)}$, and the corresponding frequency of double homozygotes is $\sum_i f_i (p_{11}^{(i)})^2$, where the summation is over all selfing ages. The pairwise identity disequilibrium, which is just the (appropriately normalized) difference between the double homozygote frequency and the square of the single-locus homozygote

frequency, is given by:

$$
\begin{aligned}
(ID)_{pair} &= \left[\sum_i f_i [p_{11}^{(i)}]^2 - \sum_i \sum_j f_i f_j p_{11}^{(i)} p_{11}^{(j)}\right] \Big/ p^2(1-p)^2 \\
&= \left[\sum_i f_i (1-f_i) [p_{11}^{(i)}]^2 - \sum_i \sum_{j \neq i} f_i f_j p_{11}^{(i)} p_{11}^{(j)}\right] \Big/ p^2(1-p)^2 \\
&= \left[\sum_i f_i \sum_{j \neq i} f_j [p_{11}^{(i)}]^2 - \sum_i \sum_{j \neq i} f_i f_j p_{11}^{(i)} p_{11}^{(j)}\right] \Big/ p^2(1-p)^2 \qquad (1) \\
&= \left[\sum_i \sum_{j<i} f_i f_j ([p_{11}^{(i)}]^2 + [p_{11}^{(j)}]^2) - 2 \sum_{j<i} f_i f_j p_{11}^{(i)} p_{11}^{(j)}\right] \Big/ p^2(1-p)^2 \\
&= \left[\sum_i \sum_{j<i} f_i f_j [p_{11}^{(i)} - p_{11}^{(j)}]^2\right] \Big/ p^2(1-p)^2
\end{aligned}
$$

where the double summation is over all possible pairs of selfing ages. The normalisation term involves $p$, the population-wide frequency if the '1' allele. Thus, positive population-wide ID emerges, as long as the average homozygosity is *different* across cohorts. A similar expression can be obtained for the pairwise LD.

The key assumption underlying the ILEC approximation is that a single generation of outcrossing erases most associations between loci, such that the cohort of outcrossed offspring is a well-mixed group with little or no population structure. This assumption would be clearly untenable if loci are linked, or if there are epistatic interactions between loci (for example, if there were strong stabilizing selection on the additive trait). Even in the present scenario involving multiplicative selection across unlinked loci, this assumption is not strictly true, as outcrossing individuals with different selfing histories have different allele frequencies and homozygosities (resulting in differences in segregation variance among different outcrossing pairs), which can generate some structure within the cohort of outcrossed offspring. Nevertheless, this approximation generates predictions for detailed attributes of large populations,

which agree quite well with results of individual-based simulations.

Under the ILEC approximation, the population is described completely by the occupancies $f_i$ of each selfing age cohort $i$, and the frequency of heterozygous and homozygous alleles for each type of locus within each cohort. Let us denote the frequencies of homozygous and heterozygous additive loci (carrying the '1' allele) within the $i^{th}$ cohort by $p^{(i)}_{11,A}$ and $p^{(i)}_{01,A}$, and the corresponding frequencies at partially recessive loci by $p^{(i)}_{11,R}$ and $p^{(i)}_{01,R}$. The evolution of $f_i$, $p^{(i)}_{11,A}$, $p^{(i)}_{01,A}$, $p^{(i)}_{11,R}$ and $p^{(i)}_{01,R}$ under mutation, selection and partial selfing can then be described using the following equations:

**Mutation**

$$\begin{aligned} p^{(i)}_{11,A} &\to (1-\mu_A)^2 p^{(i)}_{11,A} + \mu_A(1-\mu_A)p^{(i)}_{01,A} + \mu_A^2(1-p^{(i)}_{11,A}-p^{(i)}_{01,A}) \\ p^{(i)}_{01,A} &\to 2\mu_A(1-2p^{(i)}_{01,A}) + p^{(i)}_{01,A} \end{aligned} \tag{2a}$$

$$f^{(i)} \to f^{(i)} \tag{2b}$$

Equation (2a) represents the change in homozygote and heterozygote frequencies due to mutation at a single additive locus. A similar equation can be written for frequency changes at recessive loci, by replacing $\mu_A$ by $\mu_R$. The fraction of individuals in each cohort itself remains unchanged by mutation (eq. (2b)).

**Selection**

$$\begin{aligned} &p^{(i)}_{11,A} \to e^{-\tilde{s}} p^{(i)}_{11,A}/\overline{W^{(i)}_A} \qquad p^{(i)}_{01,A} \to e^{-\tilde{s}/2} p^{(i)}_{01,A}/\overline{W^{(i)}_A} \\ &\text{where} \quad \overline{W^{(i)}_A} = (1-p^{(i)}_{11,A}-p^{(i)}_{01,A}) + e^{-\tilde{s}} p^{(i)}_{11,A} + e^{-\tilde{s}/2} p^{(i)}_{01,A} \qquad \tilde{s} = \alpha s \end{aligned} \tag{3a}$$

$$p_{11,R}^{(i)} \to e^{-s} p_{11,R}^{(i)} / \overline{W_R^{(i)}} \qquad p_{01,R}^{(i)} \to e^{-hs} p_{01,R}^{(i)} / \overline{W_R^{(i)}}$$
$$\text{where} \quad \overline{W_R^{(i)}} = (1 - p_{11,R}^{(i)} - p_{01,R}^{(i)}) + e^{-s} p_{11,R}^{(i)} + e^{-hs} p_{01,R}^{(i)} \tag{3b}$$

$$f^{(i)} \to \frac{f^{(i)} \left[\overline{W_A^{(i)}}\right]^{L_A} \left[\overline{W_R^{(i)}}\right]^{L_R}}{\sum_j f^{(j)} \left[\overline{W_A^{(j)}}\right]^{L_A} \left[\overline{W_R^{(j)}}\right]^{L_R}} \tag{3c}$$

Equations (3a) and (3b) represent the effect of selection on heterozygote and homozygote frequencies at an additive locus and a recessive locus respectively, within a selfing-age cohort $i$. Equation (3c) shows how selection causes the proportions of different cohorts within the population to change in proportion to their average fitness. Fitness is determined multiplicatively by the additive and recessive loci— the contribution of a single additive locus to the average fitness of the $i^{th}$ cohort is denoted by $\overline{W_A^{(i)}}$; the contribution of all additive loci is $\left[\overline{W_A^{(i)}}\right]^{L_A}$, and similarly for recessive loci.

## Mating

$$p_{11,A}^{(i+1)} \to p_{11,A}^{(i)} + \frac{p_{01,A}^{(i)}}{4} \qquad p_{01,A}^{(i+1)} \to \frac{p_{01,A}^{(i)}}{2} \tag{4a}$$

$$p_{11,A}^{(0)} \to p_A^2 \qquad p_{01,A}^{(0)} \to 2p_A(1-p_A) \qquad \text{where } p_A = \sum_i f^{(i)} \left(p_{11,A}^{(i)} + p_{01,A}^{(i)}/2\right) \tag{4b}$$

$$f^{(0)} \to 1 - r_s \qquad f^{(i+1)} \to r_s f^{(i)} \tag{4c}$$

Equation (4a) shows how homozygote and heterozygote frequencies at an additive locus change due to selfing: note that the new frequencies within the $(i+1)^{th}$ cohort depend on the old frequencies within the $i^{th}$ cohort. Equation (4b) shows the new heterozygote and

homozygote frequencies within the outcrossing cohort: these depend only on the population-wide allele frequency $p_A$, and not on the frequencies of the additive allele within each cohort separately. Equations identical to (4a) and (4b) can be written down for frequency changes at recessive loci. Equation (4c) shows the proportion of individuals belonging to different selfing age cohorts after mating; the fraction of outcrossed individuals (with selfing age 0) is just $1 - r_s$.

Equations (2)-(4) can be iterated over generations, until the proportions $f_i$, and the homozygote and heterozygote allele frequencies $p^{(i)}_{11,A}$, $p^{(i)}_{01,A}$, $p^{(i)}_{11,R}$ and $p^{(i)}_{01,R}$ reach stationary (equilibrium) values. Note that the concept of an equilibrium or steady state is not strictly well-defined for this model: starting with a fully outcrossed population (i.e., $f_0 = 1$, $f_i = 0$ for all $i > 0$) at $t = 0$, cohorts with higher and higher selfing ages are generated in each generation. Thus, in principle, $f_i$ can be non-zero for all cohorts $i$ with selfing ages $0, 1, \cdots t$ in generation $t$. However, the proportions $f_i$ become vanishingly small for $i \gg -1/\log(r_s)$ even in the absence of selection, while $f_i$ show a steeper decline with $i$ when there is selection against partially recessive alleles. Thus, in practice, all cohorts reach equilibrium if the above recursions are iterated sufficiently long. The fact that only the first few cohorts have non-zero occupancy makes this a relatively economical way of approximating population structure in a highly polygenic context.

Under the ILEC approximation, we can express the frequency of any multi-locus genotype in terms of the proportions $f_i$ and the homozygote and heterozygote frequencies in each cohort. The probability $P(m_{11,A}, m_{01,A}, m_{11,R}, m_{01,R})$, that an individual has $m_{11,A}$ homozygous and $m_{01,A}$ heterozygous loci for the additive '1' allele, and $m_{11,R}$ and $m_{01,R}$ homozygous

and heterozygous loci respectively for the partially recessive allele, is given by:

$$P(m_{11,A}, m_{01,A}, m_{11,R}, m_{01,R}) =$$
$$\sum_i f_i \left\{ \binom{L_A}{m_{11,A}} \binom{L_A - m_{11,A}}{m_{01,A}} \quad [p_{11,A}^{(i)}]^{m_{11,A}} [p_{01,A}^{(i)}]^{m_{01,A}} [1 - p_{11,A}^{(i)} - p_{01,A}^{(i)}]^{L_A - m_{11,A} - m_{01,A}} \right.$$
$$\left. \times \binom{L_R}{m_{11,R}} \binom{L_R - m_{11,R}}{m_{01,R}} \quad [p_{11,R}^{(i)}]^{m_{11,R}} [p_{01,R}^{(i)}]^{m_{01,R}} [1 - p_{11,R}^{(i)} - p_{01,R}^{(i)}]^{L_R - m_{11,R} - m_{01,R}} \right\} \tag{5}$$

where the sum is over all selfing ages $i$ for which $f_i$ is non-zero. The equation above simply reflects the ILEC assumption that genotypes of different loci within any selfing age cohort are statistically independent of each other: then the numbers of loci (of a particular type) with states '00', '01' and '11' must have a trinomial distribution across individuals within a particular selfing age. Equation 5 allows us to calculate any pairwise associations, as in eq. (1) above. We can also use eq. (5) to generate founder genotypes when simulating colonisation from a source population. Approximate distributions of genetic load in the population under the ILEC approximation (shown by lines in figs. 2C and 2D in the main text) were obtained by first sampling a large numbers of genotypes according to eq. (5) and then plotting the distribution of load among these.

# S2. Testing the validity of ILEC approximation for highly selfing populations.

The ILEC approximation is a deterministic approximation, which is expected to break down when drift overwhelms selection, i.e., for $N_e s < 1$. The effective population size $N_e$ decreases sharply as the selfing fraction approaches 1, especially if the genome-wide mutation rate is high and/or selection on deleterious variants weak (Kamran-Disfani and Agrawal ,

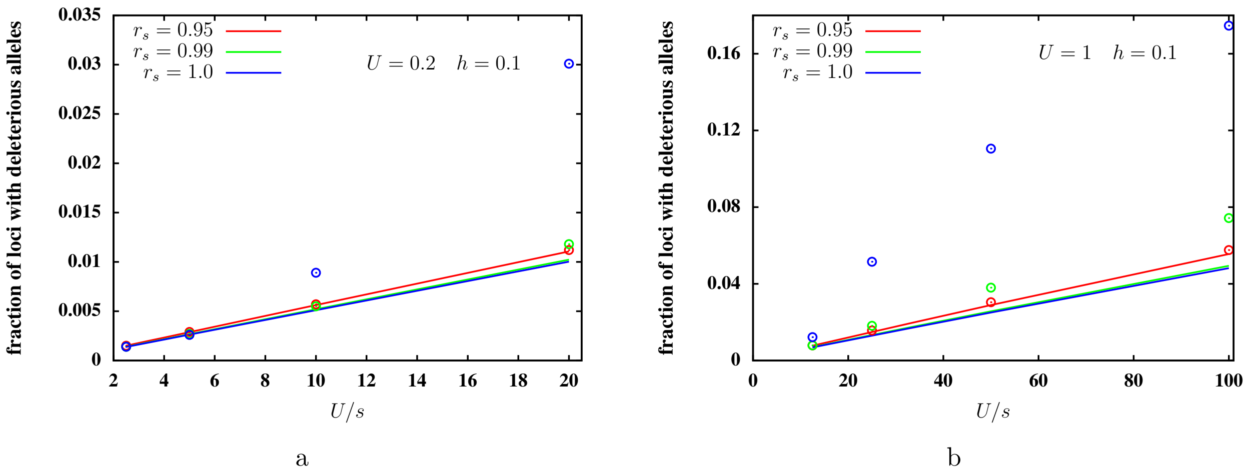


Figure 1: Comparison of individual-based simulations with ILEC predictions for high selfing fractions ($r_s = 0.95$, $0.99$ and $1$), for genome-wide mutation rate (A) $U = 0.2$ and (B) $U = 1$. Points depict the fraction of loci with deleterious alleles, as found in individual-based simulations after evolving each population for 2000 generations from a pre-specified initial state; lines depict the corresponding ILEC prediction for the equilibrium frequency of the deleterious allele. Parameters used: $N = 10^4$, $L = 1000$, $h = 0.1$. Each point is obtained by averaging over 100 replicate populations. The range of $U/s$ values over which the ILEC approximation holds becomes more restricted as we approach $r_s = 1$.

2014). This allows weakly deleterious alleles to drift close to fixation; then the average number of deleterious alleles per genome is significantly higher than the corresponding ILEC prediction (note the fraction of loci with deleterious alleles with $s = 0.01$ for $r_s = 0.95$, compared to the ILEC prediction in figs. 1A and 1B in the main paper).

Here, I use individual-based simulations with $N = 10000$ individuals to further investigate the accuracy of the ILEC approximation for a range of $U/s$ values, for $r_s = 0.95, 0.99, 1$. For simplicity, all loci are assumed to be identical. Populations are initialized to be in Hardy-Weinberg and linkage equilibrium, with the frequency of the deleterious allele per locus set equal to the ILEC prediction. Population are then evolved for 2000 generations, and the fraction of loci with deleterious alleles plotted against the corresponding ILEC prediction (fig. 1). Note that for $U/s \gg 1$ and $r_s \sim 1$, populations may not necessarily be at equilibrium after 2000 generations (as they undergo a steady buildup of deleterious alleles).

For the parameters used in fig. 1, the ILEC approximation is valid for all depicted values of $U/s$ in populations with $r_s = 0.95$. However, the approximation breaks down for $U/s \gtrapprox 20$ for $r_s = 0.99$, and $U/s \gtrapprox 10$ for $r_s = 1.0$. This suggests that in these regimes, $N_e s$ is reduced well below 1 even for populations with $Ns$ as large as 100 and no linkage between deleterious variants. However, a general understanding of the effect of background selection on $N_e$ in highly selfing populations with selective interference between weakly deleterious variants remains lacking. In particular, approximations that neglect linkage disequilibria between loci (e.g., Glémin and Ronfort (2013)) may overestimate $N_e$ close to $r_s = 1$ (Kamran-Disfani and Agrawal , 2014).

Note that our model assumes bidirectional mutation (i.e., allows for reverse mutation from the deleterious state), as opposed to unidirectional mutation. This choice makes very little quantitative difference to most of the results, except when selfing fractions are close to 1. For $r_s \sim 1$, the number of deleterious mutations per genomes increases even faster when there is unidirectional mutation.

# S3. Establishment with adaptive response to environment-dependent selection: scenarios with $e^{r_0}\overline{W}_{founders} > 1$.

Here, I consider establishment scenarios where fitness in the new habitat depends only on additive loci under environment-dependent selection. As shown in fig. 4B in the main paper, the establishment probability in the new habitat depends only on the mean founder fitness for sufficiently positive values of $e^{r_0}\overline{W}_{founders} - 1$, and is independent of other details such as the initial frequency of adaptive alleles or the selfing fraction. This suggests that for sufficiently high founder fitness in the new habitat, initial establishment can be approximated by a branching process, wherein a single founder produces a Poisson distributed number of

offspring with mean given by $e^{r_0}\overline{W}_{founders}$, and that each of these offspring in turn produce a Poisson-distributed number of offspring with the same mean.

Then the probability of establishment for $N_0 = 1$ can be approximated by the probability of non-extinction of a branching process with mean number of descendants $g = e^{r_0}\overline{W}_{founders}$. Let us denote the probability that the branching process goes extinct by $Q_1(g)$. From the standard theory of branching processes, it follows that this satisfies $Q_1(g) = \exp[-Q_1(g)(1-g)]$, which can be solved numerically to obtain the the extinction probability $Q_1(g)$, and hence $P_{est} = 1 - Q_1(g)$. We can also calculate $P_{est}$ for $N_0 > 1$ founders, by stipulating that the population establishes as long as at least one founder has a line of descendants that does not go extinct. This happens with probability $P_{est} = 1 - Q_1^{N_0}(g)$, under the assumption that different founders and their lineages do not interact, e.g., via shared density regulation. Establishment probabilities obtained using the branching process approximation are depicted via dashed lines in fig. 4B of the main paper, and match well with the results of individual-based simulations for $e^{r_0}\overline{W}_{founders} \gtrapprox 1.05$ for strong selection per locus, and $e^{r_0}\overline{W}_{founders} \gtrapprox 1.12$ for weak selection per locus.

A key assumption underlying the branching process approximation is that all individuals have the same number of descendants on average. Thus, this approximation neglects the effects of both segregation (which results in non-zero variance in offspring fitness around mid-parent fitness) and adaptation (which results in an increase in the average population fitness under selection). The fact that the branching process approximation nevertheless predicts establishment probabilities accurately, suggests that adaptation plays very little role during initial establishment when founder fitness is high.

# References

Glémin, S., and J. Ronfort. 2013. Adaptation and maladaptation in selfing and outcrossing species: new mutations versus standing genetic variation. Evolution 67:225-240.

Kelly, J. K. 2007. Mutation-selection balance in mixed mating populations. J. Theor. Biol. 246:355-365.

Kamran-Disfani, A., and A. F. Agrawal. 2014. Selfing, adaptation and background selection in finite populations. J. Evol. Biol. 27:1360-1371.
